# Ex-vivo changes in amino acid concentrations from blood stored at room temperature or on ice: implications for arginine and taurine measurements

**DOI:** 10.1186/1472-6890-9-10

**Published:** 2009-11-27

**Authors:** Joshua S Davis, Christabelle J Darcy, Kim Piera, Yvette R McNeil, Tonia Woodberry, Nicholas M Anstey

**Affiliations:** 1 International Health Division, Menzies School of Health Research and Charles Darwin University, Darwin, NT 0810, Australia; 2 Division of Medicine, Royal Darwin Hospital, Darwin, NT, 0810, Australia

## Abstract

**Background:**

Determination of the plasma concentrations of arginine and other amino acids is important for understanding pathophysiology, immunopathology and nutritional supplementation in human disease. Delays in processing of blood samples cause a change in amino acid concentrations, but this has not been precisely quantified. We aimed to describe the concentration time profile of twenty-two amino acids in blood from healthy volunteers, stored at room temperature or on ice.

**Methods:**

Venous blood was taken from six healthy volunteers and stored at room temperature or in an ice slurry. Plasma was separated at six time points over 24 hours and amino acid levels were determined by high-performance liquid chromatography.

**Results:**

Median plasma arginine concentrations decreased rapidly at room temperature, with a 6% decrease at 30 minutes, 25% decrease at 2 hours and 43% decrease at 24 hours. Plasma ornithine increased exponentially over the same period. Plasma arginine was stable in blood stored on ice, with a < 10% change over 24 hours. Plasma taurine increased by 100% over 24 hours, and this change was not prevented by ice. Most other amino acids increased over time at room temperature but not on ice.

**Conclusion:**

Plasma arginine concentrations in stored blood fall rapidly at room temperature, but remain stable on ice for at least 24 hours. Blood samples taken for the determination of plasma amino acid concentrations either should be placed immediately on ice or processed within 30 minutes of collection.

## Background

Quantification of plasma amino acids is not routinely offered by clinical laboratories and thus plasma often needs to be transported to research or reference laboratories for testing. In order to accurately assess the concentration of plasma amino acids, it is important to know their stability in human blood which has been stored or transported prior to testing. Previous studies addressing this question have been small and the rate of degradation has not been precisely quantified.

Arginine, the precursor of nitric oxide (NO) [1], is important for endothelial [2] and immunological [3] function and is acutely decreased in sepsis [4,5], malaria [6] and trauma [7], and was thus the focus of this study. The major routes for arginine metabolism in humans are metabolism by arginase to urea and ornithine; use for creatine synthesis; and metabolism by nitric oxide synthase to NO and citrulline [8]. Both red blood cells (RBCs) [9] and macrophages [10] are rich in arginase. In stored packed RBCs, arginase is released and the resulting degradation of plasma arginine is thought to be a mechanism of transfusion-associated immunosuppression [9,11]. Other amino acids which are commonly added to supplementary nutrition for critically ill patients may also play an important role in immune function including tryptophan [12] glutamine [13] and taurine [14,15].

Hainque and colleagues studied eight healthy volunteers and found a "significant degradation" of plasma arginine following 4 hours at room temperature but this was not quantified and no other time points were reported [16]. Schaefer et al. studied one volunteer and found a 50% decrease in plasma arginine after 6 hours at room temperature compared with a 10% decrease after 6 hours at 4 degrees centigrade, with earlier time points not reported [17]. Nutall and colleagues reported time profile data from one volunteer, which showed an approximate 33% decrease in plasma arginine by 2 hours at room temperature [18].

To determine the impact of delayed processing we undertook a study to estimate the rate of arginine degradation in human plasma at room temperature and on ice. We hypothesised that this degradation would be primarily due to plasma arginase activity and that there would be less than 10% degradation at 2 hours in samples placed immediately on ice. We also sought to determine the effect of delayed separation and freezing of plasma on the concentration of other amino acids.

## Methods

The study was considered by the Chair of the Human Research Ethics Committee of the Menzies School of Health Research and Northern Territory Department of Health and Families, and was approved as a laboratory quality assurance activity which did not require full ethical review. Following written informed consent, six healthy normotensive fasting volunteers had venous blood collected into 12 × 2 mL lithium heparin tubes (Vacutainer, Becton Dickinson, Franklin Lakes, New Jersey) using a 21 gauge needle and vacutainer system. For each subject, the first six tubes were immediately placed into an ice slurry and the second six were left at room temperature (25° Celsius (C)) in an air conditioned laboratory. After intervals of 0 minutes, 30 minutes, 2 hours, 4 hours, 8 hours and 24 hours from the time of venepuncture, the tubes were centrifuged at 3000 rpm for 10 minutes (either at 4°C or at room temperature as appropriate) and the plasma immediately separated and stored at -80°C.

Subsequently, following thawing, plasma amino acids were extracted with ethanol, then derivatized with AccQ-Fluor (Waters, Milford, MA). Amino acid concentrations were then determined by reverse-phase high performance liquid chromatography (HPLC; Shimadzu corporation, Kyoto, Japan) with UV (250 nm) and fluorescence (excitation 250 nm, emission 395 nm) detection, using a method modified from van Wandelen and Cohen [19].

The data were analysed using Stata 10 (Statacorp, College Station, Texas) and GraphPad Prism 5 (Graphpad software, San Diego, California). Due to the small number of subjects, data were summarized using median and interquartile range. Median amino acid concentrations over time were compared using a paired Wilcoxon test, with a p-value of < 0.05 considered significant. The arginine degradation curve was fitted using a one-phase exponential decay model. The sample size was determined using data from an earlier experiment (unpublished data), which found that there was 31.8% (std dev = 14%) degradation of arginine at room temperature by 2 hours. Using a power of 80% and a significance level of 5%, five subjects in each group would be needed to detect a difference of 22% degradation at 2 hours, meaning less than 10% degradation in the ice group. To allow for sample wastage and errors, we recruited six subjects.

## Results

Of the six study subjects, half were male, and the median age was 37.5 years, with a range of 19-47 years (table 1). All were healthy, of normal weight and normotensive, and none had cardiovascular disease or diabetes mellitus. The median baseline plasma arginine concentration was 74.9 μmol/L, similar to previously reported mean plasma arginine concentrations from healthy volunteers, the majority of which are between 60 and 80 μmol/L [20].

**Table 1:** Characteristics of study subjects

Subject	Age (years)	Gender	Ethnicity
**1**	36	F	Caucasian

**2**	39	M	Caucasian

**3**	47	F	Caucasian

**4**	27	F	Caucasian

**5**	19	M	Caucasian

**6**	44	M	Caucasian

### Arginine and ornithine time profiles at room temperature

**Figure 1:**
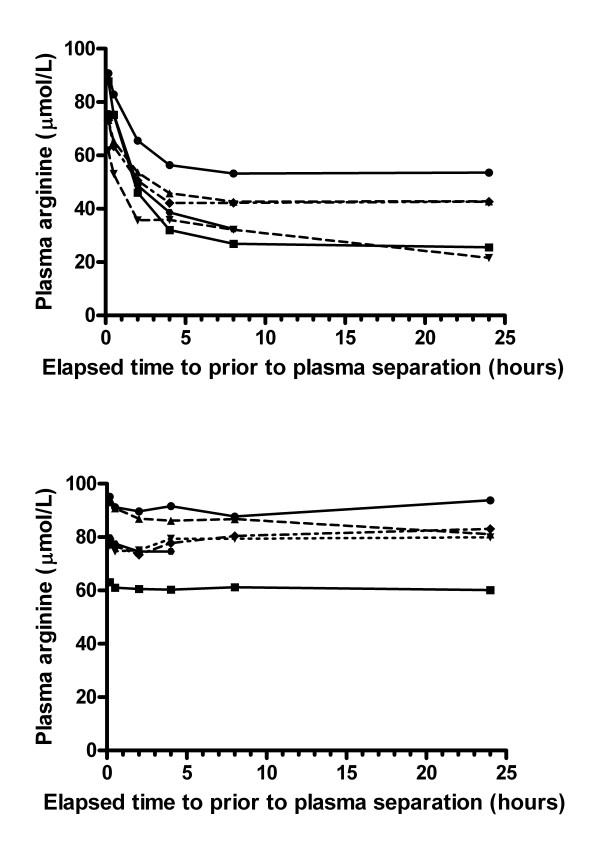
**Plasma arginine time profile at room temperature and on ice**. Each curve represents an individual subject. Figure 1a depicts results from whole blood stored at room temperature (25°C). Figure 1b depicts results from aliquots of the same blood samples which were stored in an ice slurry.

**Figure 2:**
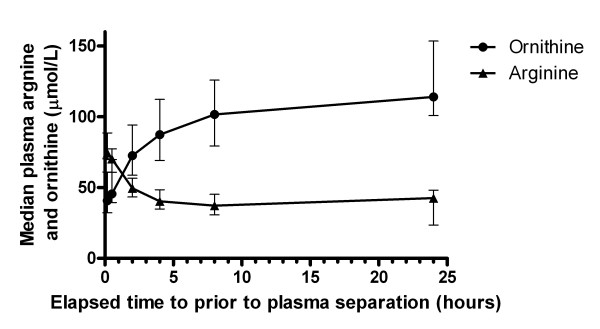
**Time profile of median plasma arginine and ornithine concentrations in blood stored at room temperature**. Each point represents the median value for that time, and the error bars represent the interquartile range. Median plasma arginine is indicated by triangles, and ornithine by solid circles.

Plasma arginine concentration decreased rapidly at room temperature (Figures 1a, 2, Table 2) with 6% degradation within 30 minutes, 25% degradation within 2 hours and 43% degradation within 24 hours. A non-linear model of the plasma arginine profile over time was defined by the equation Y = ((Y0-P)*e^-kt^)+P, where t = time in hours, P = the plateau value, Y0 = initial value. The parameters of the model were Y0 = 81.3, P = 37.8, and k = 0.6273. This model fitted the data well, with an R^2 ^of 0.73. Plasma ornithine concentration increased exponentially at room temperature (Table 1, Figure 2), with a 4% increase at 30 minutes, a 62% increase at 2 hours, and a 183% increase at 24 hours.

**Table 2:** Median (IQR) arginine and ornithine plasma concentrations over time from blood stored at room temperature compared with stored on ice

	Baseline	30 minutes	2 hours	4 hours	8 hours	24 hours
**Arginine RT^a^**	74.9	70.3	49.6	40.4	37.3	42.6

	73.2-87.8	63.4-75.5	46.0-53.6	35.8-45.8	32.1-42.6	25.5-42.8

**Arginine Ice**	79.6	77.1	74.8	78.6	80.4	81.0

	76.8-93.0	74.6-90.8	73.4-86.9	74.6-86.1	79.4-86.7	79.9-83.0

**Ornithine RT**	44.7	45.6	72.6	87.4	101.6	114.1

	32.9-60.8	39.5-69.8	58.7-94.2	69.1-112.3	79.4-125.9	100.9-153.6

**Ornithine Ice**	38.6	31.6	39.2	40.5	36.3	43.1

	29.4-57.3	38.3-59.2	30.2-60.0	30.5-61.9	32.8-61.5	36.2-68.1

a. RT = Room Temperature

### Arginine time profile on ice compared with room temperature

Plasma arginine was very stable on ice, with a less than 10% change over a 24 hour period. At 2 hours, the median plasma arginine concentration had decreased by 6% in the ice specimens compared with 25% in the room temperature specimens (p < 0.001) (Figure 1). At 24 hours, the change in arginine was negligible for the ice specimens compared with a 43% decrease at room temperature (p < 0.001). Ornithine was also more stable on ice, with a 24% increase over the 24 hour period, compared with a 183% increase at room temperature.

### Time profile of other amino acids

**Figure 3:**
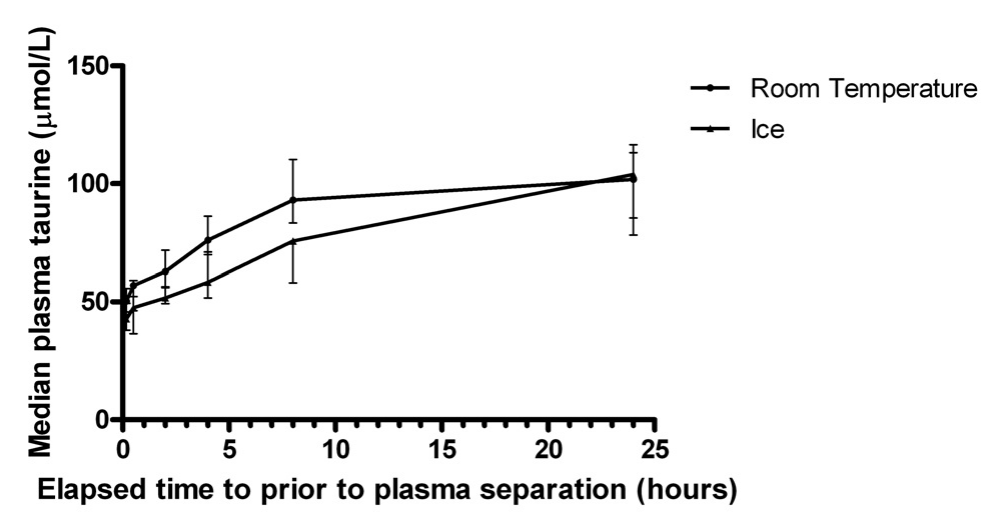
**Time profile of median plasma taurine concentrations in blood stored at room temperature and on ice**. Each point represents the median value for that time, and the error bars represent the interquartile range. Median plasma taurine at room temperature is represented by solid circles, and median plasma taurine on ice is represented by triangles.

For the majority of other amino acids, concentrations increased by > 10% over 24 hours at room temperature (Table 3). The majority of these changes were largely or completely prevented in the blood that was placed on ice. The most notable room temperature concentration increases at 24 hours were seen with taurine (which doubled) and glutamate (which increased more than fivefold). The change in taurine was unusual in that it was more marked in the blood placed on ice (a 126% increase) than the room temperature specimens (a 100% increase), suggesting that the increase in taurine may be due to release from lysed cells rather than to an enzymatic process (Figure 3). Tryptophan was very stable both at room temperature and on ice.

**Table 3:** Change in amino acid concentrations in whole blood after 24 hours at room temperature and on ice.

	% Change at 24 h at RT^a, b^	% Change at 24 h on ice^b^
***Group 1 - ≤ 10% change at RT^a ^over 24 h***		

**Citrulline**	-4 (-6, 4)	-7 (-9, -4)

**Glutamine**	-10 (-13, -10)	-5 (-5, -4)

**Hydroxyproline**	8 (7,9)	-3 (-4,-3)

**Methionine**	0 (-2, 1)	1 (1, 6)

**Tryptophan**	7 (5, 8)	4 (4, 6)

**Tyrosine**	8 (5, 12)	-2 (-3, -1)

**Valine**	8 (4, 11)	1 (0, 1)

***Group 2 - > 10% increase at RT^a ^over 24 h***		

**Alanine**	18 (16,20)	0 (-1, 0)

**Asparagine**	17 (12, 21)	0 (-1, +3)

**Glutamate**	593 (563, 612)	38 (92, 186)

**Glycine**	26 (24, 34)	3 (2, 4)

**Histidine**	23 (17, 27)	1 (0, 1)

**Isoleucine**	16 (10, 21)	0 (-1, 2)

**Leucine**	23 (17, 34)	2 (1, 5)

**Lysine**	19 (18, 19)	2 (1, 5)

**Ornithine**	183 (180, 224)	24 (23,25)

**Phenylalanine**	15 (14,22)	1 (1, 3)

**Proline**	11 (6,13)	1 (-1,2)

**Serine**	18 (17,28)	6 (2,6)

**Taurine**	100 (94, 102)	126 (120, 147)

**Threonine**	11 (10, 14)	-2 (-5, 0)

***Group 3 - > 10% decrease at RT^a ^over 24 h***		

**Arginine**	-43 (-65, -43)	-1 (-5, 4)

a. RT - Room temperature

b. Expressed as median % change (Interquartile range)

Note - % change values are an increase (positive change) unless otherwise specified.

## Discussion

Plasma arginine concentration decreases rapidly in whole blood held at room temperature, and this decrease is greatly attenuated by placing the blood on ice. Ornithine, the metabolic product of arginine metabolism by arginase, rises exponentially at room temperature, and this rise does not occur on ice, suggesting that it is due to an enzymatic process. Thus, it is likely that arginase is the primary mechanism of arginine degradation in ex-vivo blood samples. This arginase could come from either lysed RBCs or lysed leucocytes, but we did not evaluate the source of arginase, and thus cannot determine which of these was more important. In-vitro hemolysis is difficult to measure, as the released cell-free haemoglobin is immediately bound by haptoglobin. While we have not proven this hypothesis, our observations strongly suggest it.

Most other amino acids increase at room temperature but not on ice, which also implies an enzymatic reaction. Tryptophan is very stable both at room temperature and on ice. Taurine and glutamine are unusual, in that they increase markedly both at room temperature and on ice; this may be due to cellular release rather than enzymatic catabolism.

The rate of decrease of plasma arginine which we found in blood held at room temperature is similar to that found by Nuttall and colleagues in the only published paper to have reported plasma arginine concentrations at room temperature at more than two time points [18]. The lack of early time points in other papers makes it difficult to estimate the rate of decline and whether it is linear or exponential. Nuttall et al. reported data in graphical form, from a single subject up to 2.5 hours post venepuncture. They found a fall from 89 μmol/L to approximately 60 μmol/L at 2 hours (a 33% drop), similar to our reported decrease of 25% at 2 hours.

The large increases seen in taurine and glutamate in our study have not previously been reported. Sahai et al. measured amino acid levels in whole blood from twenty-two volunteers, stored on ice for 1 hour or 2 hours, and found a less than 10% decrease in plasma taurine and glutamate at 1 and 2 hours [21]. Shaeffer et al. reported a < 10% decrease in plasma taurine and glutamate at 6 hours in blood held at room temperature from one healthy volunteer [17]. The reason for this discrepancy is unclear. Both papers used different methods for amino acid quantification than we did. Sahai et al did not measure time points beyond 2 hours, and most of the increase in both taurine and glutamine in our study occurred beyond 2 hours. However, until this finding is reproduced by other investigators, it should be regarded with caution.

The primary limitations of this study are the relatively small number of subjects and the lack of subjects suffering from sepsis, trauma or other conditions of interest. A larger number of subjects would allow a more accurate estimate of the time profile of arginine degradation over time. Considering arginase activity is increased in severe sepsis [22] and trauma [23], it is unclear if blood from patients with these conditions would yield the same results as we observed. We did not directly measure arginase activity in blood or plasma, and thus our inference that plasma arginase is primarily responsible for the observed ex-vivo arginine degradation is based on indirect evidence. However, the only other significant mechanism for arginine degradation likely to occur ex-vivo is the breakdown of arginine to NO and citrulline by nitric oxide synthase, which accounts for less than 5% of arginine metabolism in healthy humans [24].

One potential implication of these data is that whole blood stored for the purpose of transfusion is likely to contain non-physiological concentrations of amino acids, which may have unintended immunosuppressive effects. These data also reinforce the importance of accurate methodological descriptions in papers reporting plasma amino acid levels. In a hospital setting, it is not always possible to process samples within 30 minutes of collection. It is therefore essential to note the time between collection and freezing when reporting concentrations of plasma amino acids. This is particularly important if the sample cannot be kept on ice - for example, if the blood is to be used for both peripheral blood mononuclear cell (PBMC) collection and amino acid analysis. As PBMCs are damaged by freezing, these samples must be kept at room temperature and processed as soon as possible to allow accurate analysis of both PBMC function and amino acid concentrations. Furthermore, where plasma amino acids are being measured for clinical applications, our data emphasise the importance of timely separation and freezing of plasma to avoid potential diagnostic errors.

## Conclusion

In conclusion, arginine undergoes rapid ex-vivo degradation at room temperature but this does not occur on ice; plasma tryptophan is stable for at least 24 hours both at room temperature and on ice; plasma taurine concentrations show large increases both at room temperature and on ice. Blood collected for the purposes of plasma amino acid determination should be placed immediately on ice; if this is not possible, plasma should be frozen with 30 minutes of collection.

## Abbreviations

HPLC: High Performance Liquid Chromatography; NO: Nitric Oxide; PBMC: Peripheral Blood Mononuclear Cell; IQR: Interquartile range.

## Competing interests

The authors declare that they have no competing interests.

## Authors' contributions

All authors took part in study design and contributed to the final draft of the paper. In addition, JSD participated in interpretation of HPLC results, performed the data analysis and wrote the first draft of the paper. CJD, KP, and TW performed sample preparation. YM performed and analysed the HPLC. NA secured the funding. All authors read and approved the final manuscript.

## Funding sources

The study was funded by the National Health and Medical Research Council of Australia (NHMRC Program Grants 290208, 496600; Practitioner Fellowship to NMA, Scholarship to JSD). The funders had no role in study design, data collection and analysis, decision to publish, or preparation of the manuscript.

## Pre-publication history

The pre-publication history for this paper can be accessed here:

http://www.biomedcentral.com/1472-6890/9/10/prepub
